# Effect of a Low Concentration of Sevoflurane Combined With Propofol on Transcranial Electrical Stimulation Motor Evoked Potential: A Case Series

**DOI:** 10.7759/cureus.41562

**Published:** 2023-07-08

**Authors:** Shoto Yamada, Tomohiro Chaki, Yusuke Kimura, Nobuhiro Mikuni, Michiaki Yamakage

**Affiliations:** 1 Division of Clinical Engineering, Sapporo Medical University Hospital, Sapporo, JPN; 2 Department of Anesthesiology, Sapporo Medical University, Sapporo, JPN; 3 Department of Neurosurgery, Sapporo Medical University, Sapporo, JPN

**Keywords:** intraoperative neurophysiological monitoring (ionm), propofol, sevoflurane, transcranial electrical stimulation, motor evoked potential monitoring

## Abstract

Transcranial electrical motor evoked potential (TCeMEP) is used to monitor the integrity of intraoperative motor function. Total intravenous anesthesia (TIVA) is the preferred method because its effect on MEP is relatively smaller than volatile anesthetics. However, maintaining the balanced anesthesia in long-time surgery using TIVA is challenging and may sometime cause problems including body movement during microsurgery. Such problems can be avoided by intraoperative anesthesia management using a mixture of propofol and a low concentration of sevoflurane. We recorded TCeMEP under a mixture of propofol and low concentration of sevoflurane anesthesia in three cases of neurosurgery. Anesthesia was induced with a 5.0 µg/mL target-controlled infusion of propofol and 0.6 mg/kg rocuronium. General anesthesia was maintained by propofol and 0.1-0.25 µg/kg/min remifentanil infusion. After the recording of control TCeMEP, sequential inhalation of 0.2 minimum alveolar concentration (MAC) and 0.5 MAC of sevoflurane was performed. The duration of each sevoflurane inhalation was 10 minutes, and the MACs were adjusted by the patient’s age. In our cases, the combination of propofol and 0.2 MAC sevoflurane suppressed the amplitude of TCeMEP to 38.0±21.7% (379.8±212.0 µV), but the amplitude was high enough for evaluation of motor function monitoring. On the other hand, the combination of 0.5 MAC sevoflurane greatly decreased the amplitude of TCeMEP to 6.3±6.0% (71.9±66.9 µV) resulting in less than 150 µV, and it was difficult to record the change in TCeMEP amplitude over time. The combination of 0.2 MAC sevoflurane with TIVA might enable TCeMEP monitoring with TIVA.

## Introduction

Intraoperative neurophysiological monitoring (IONM) using motor-evoked potentials (MEPs) with direct cortical stimulation or transcranial electrical stimulation (TCeMEP) has been widely used in orthopedics and neurosurgery [1,2]. It has been reported that the inhalational anesthetics including sevoflurane have made intraoperative MEP recording difficult [3]. However, the use of total intravenous anesthesia (TIVA) with propofol and opioids and the development of high-frequency pulsed MEP stimulation techniques have led to successful IONM of the corticospinal tract under general anesthesia [1,4,5]. Propofol causes less suppression of I-waves in lower motor neurons of the spinal cord than does an inhalational anesthetic [6,7]. The anesthetic management using TIVA and these stimulation techniques has become the standard in intraoperative MEP recording. However, maintaining the balanced anesthesia in long-time surgery using TIVA is challenging and may sometime cause problems including body movement during microsurgery [8]. To prevent the problem, anesthetic management using the combination of propofol and a low concentration of sevoflurane might be effective [8], but the influence on IONM has not been well elucidated. In IONM, because MEP has high versatility and usefulness, it has clinical significance to know this influence. We report three cases in which changes over time of TCeMEP could be observed under anesthetic management using propofol and a low concentration of sevoflurane in neurosurgery.

## Case presentation

Written informed consent was obtained from all patients on the day before surgery. TCeMEP was monitored in three patients undergoing neurosurgery in whom the monitoring was required for preventing surgery-induced impairment of motor function. Details of the patients’ profiles are shown in Table 1.

**Table 1:** Patient characteristics SI: Stimulation intensity, TCeMEP: Transcranial electrical motor evoked potential

Case	Age	Sex	Height [cm]	Weight [kg]	Diagnosis	Duration [min]		TCeMEP stimulation [mA]	
						Surgery	Anesthesia	Threshold	SI
1	46	F	158.3	51.7	Glioma	418	540	95	115
2	59	F	162.0	40.0	Metastasis cerebellar tumor	405	564	58	75
3	32	F	163.7	54.8	Internal carotid artery aneurysm	480	605	70	90

General anesthesia

All anesthetic procedures were unified according to the institutional guidelines. Each of the patients entered the operating room without premedication. Before induction of general anesthesia, American Society of Anesthesiologists standard monitors including a monitor for non-invasive blood pressure, electrocardiogram, and pulse oximetry were placed. Anesthesia was induced with a 5.0 µg/mL target-controlled infusion of propofol and 0.6 mg/kg rocuronium. After tracheal intubation, general anesthesia was maintained by propofol and 0.1−0.25 μg/kg/min remifentanil infusion, and the infusion rate of propofol was adjusted to achieve a bispectral index value of 40−60 until the end of the surgical procedure including the period of sevoflurane mixture inhalation. After placement of TCeMEP electrodes and recording of control TCeMEP, neuromuscular blockade was antagonized with sugammadex, and sequential inhalation of 0.2 and 0.5 minimum alveolar concentrations (MACs) of sevoflurane, which has a weak muscle relaxant effect, was performed. The duration of each sevoflurane inhalation was 10 min and the MACs were adjusted by the patient’s age [9]. After the TCeMEP recording, sevoflurane inhalation was discontinued, and general anesthesia was maintained with propofol and remifentanil infusion.

Assessment and measurement of TCeMEP

We confirmed the central sulcus using a navigation system (Curve Navigation, Brainlab AG, Feldkirchen, Germany) and, according to the International 10-20 system, placed the two corkscrew electrodes on the skull as C3 and C4. TCeMEP was recorded from the abductor pollicis brevis and abductor halluces muscles using a pair of surface electrodes placed 3 cm apart in each belly and tendon. TCeMEP was derived as compound muscle action potential after monophasic electrical stimulation with the anode of C3 or C4 on the affected side and the cathode of C4 or C3 on the pair side electrodes. All TCeMEP stimulations and recordings were performed on a Digitimer Multipulse Stimulator (Neuromaster MEE-2000, Nihon Kohden, Tokyo, Japan). The stimulation waveform was obtained by a constant current of rectangular pulse electrical stimulus intensity (1−200 mA). Other stimulus conditions were as follows: pulse duration of 0.3 ms, inter-stimulus interval of 2.0 ms, five train stimulations, band pass filter of 10−3000 Hz, and time base of 100 ms [10]. The TCeMEP was measured under the same settings for all the stimulation conditions except stimulus intensity. The stimulus intensity was increased stepwise increased by 1−10-mA increments from 40 mA up to a maximum of 200 mA until TCeMEP thresholds were confirmed. According to the previous study, we defined waveforms with more than 20−30 µV amplitude as TCeMEP [11], and the threshold was defined as the stimulus intensity when a waveform of 20−30 µV could be derived at a frequency of 50%. In suprathreshold stimulation (stimulus intensity of threshold + 20−30%), amplitudes of TCeMEP were measured peak-to-peak between consecutive largest peaks of positive and negative polarity. The TCeMEP was determined as positive when the MEP amplitude was stable at more than 150 µV [12]. After threshold recording, TCeMEP stimulations were performed every 30−60 seconds. The data are shown as means ± standard deviation (SD).

Case

**Figure 1:**
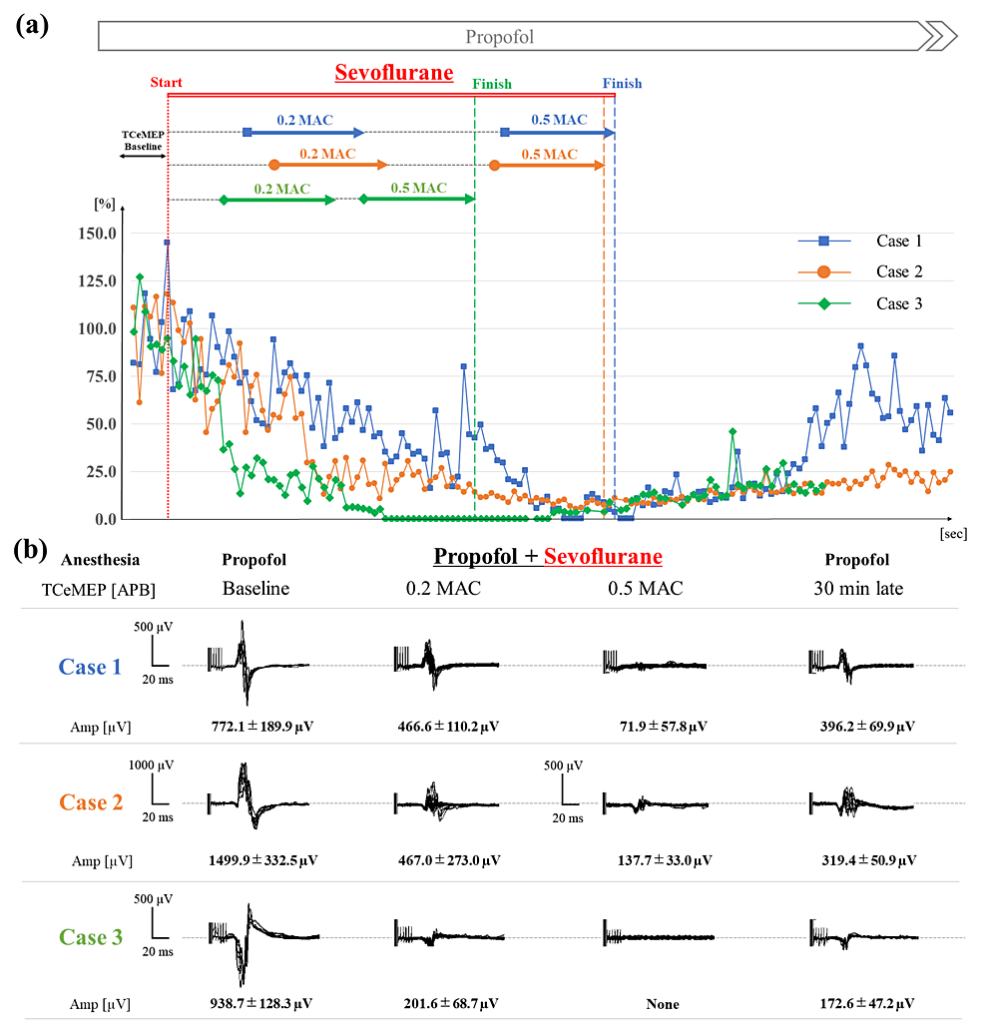
Time courses of transcranial electrical motor evoked potential (TCeMEP) amplitude in three cases (a) The chronological changes of TCeMEP under propofol and low-concentration sevoflurane anesthesia in three cases. The amplitude of TCeMEP with the baseline (propofol) was shown in percentage and plotted every 30 seconds. (b) Waveforms of TCeMEP. The baseline amplitude was 1070.2±388.2 µV. The amplitude was decreased to 38.0±21.7% (379.8±212.0 µV) by 0.2 minimum alveolar concentration (MAC) sevoflurane but remained high enough for motor function monitoring. The amplitude was greatly decreased by 0.5 MAC of sevoflurane (6.3±6.0%, 71.9±66.9 µV), and motor function monitoring was difficult in all three cases. Seven consecutive waveforms and the amplitude (mean ± standard deviation) of TCeMEP are presented. Amp: amplitude, APB: abductor pollicis brevis, MAC: minimum alveolar concentration, TCeMEP: transcranial electrical motor evoked potential

TCeMEP under the condition of propofol infusion was recorded well in all cases and the mean amplitude was 1070.2±388.2 µV. The amplitude of TCeMEP was suppressed to 38.0±21.7% (379.8±212.0 µV) by 0.2 MAC sevoflurane mixture but remained high enough for motor function monitoring. On the other hand, the amplitude was greatly decreased to 6.3±6.0% (71.9±66.9 µV) by 0.5 MAC of sevoflurane and the evaluation of TCeMEP became impossible (Figure 1). The amplitude was recovered to some extent after discontinuation of sevoflurane inhalation but did not increase to the TCeMEP baseline until the end of the measurement (29.4±17.2%, 286.2±118.1 µV). Because these changes in TCeMEP were observed before the intracranial operation, the cause of the amplitude depression was judged to be a mixture of propofol and a low concentration of sevoflurane. There was no case showing a deficit in motor function postoperatively.

## Discussion

In this case series, the combination of a low concentration of sevoflurane and propofol-based anesthesia management was used and the changes in TCeMEP over time were observed. The amplitudes decreased in all cases, but evaluation of intraoperative motor function may be possible under anesthesia using the combination of propofol and 0.2 MAC sevoflurane.

Anesthetics have almost no effect on the D-wave, but inhalational anesthetics and propofol have been reported to decrease the amplitude of the I-wave by suppressing interneurons of the motor cortex [1,6,13]. An inhalational anesthetic not only increases the activity of the γ-aminobutyric acid receptor but also reduces the activity of the N-methyl-D-aspartate receptor, and it has been shown that the suppressant effect changes progressively [14]. In contrast, propofol had less suppression of lower motor neurons than did an inhalational anesthetic, and TCeMEP amplitude under the condition of propofol infusion was significantly higher than that under the condition of sevoflurane infusion [6,7]. In addition, it has been recommended that the plasma concentration of propofol be maintained at 120−200 µg/kg/min, and that concentration hardly influences the monitoring of MEP [15]. Therefore, anesthetic regimens using propofol and an opioid (TIVA) are most suitable for intraoperative MEP [5].

For intraoperative TCeMEP, there are several studies examining anesthesia management of a mixture of propofol and inhalational anesthetic, but there was no description of a chronological or continuous change [16,17]. In such combined anesthesia, how much permits the amplitude depression of MEP is a problem. As in previous studies, a decrease in TCeMEP amplitude was observed in our cases with a low concentration of sevoflurane and was particularly remarkable at 0.5 MAC sevoflurane [16,17]. When MEP is performed while using muscle relaxant, evaluation of MEP is considered possible if there is an amplitude of more than 150 µV [12,18]. In our cases, because the combination of propofol and 0.5 MAC sevoflurane greatly decreased the amplitude of TCeMEP to less than 150 µV, the addition of 0.5 MAC sevoflurane to propofol might make it difficult to record the change in TCeMEP amplitude over time.

Inhalational anesthetics inhibit MEP monitoring, and general anesthesia thus needs to be maintained by TIVA without neuromuscular blockade. Since propofol has no analgesic or neuromuscular effects, intraoperative awareness and unanticipated movement are more likely in TIVA management. Intraoperative awareness causes posttraumatic stress disorder, and body movement may cause unexpected brain injury or cervical spinal cord injury because the patient’s head is fixed by pinning [8,19]. Therefore, intraoperative awareness and unanticipated body movement should be avoided, especially in neurosurgery. In our cases, TCeMEP monitoring could be performed under TIVA combined with 0.2 MAC of sevoflurane which provided a weak muscle relaxation effect. However, it remains unclear whether the addition of 0.2 MAC sevoflurane can decrease the incidence of intraoperative body movement. We are planning to verify the efficacy and safety of combining TIVA with a low concentration of sevoflurane in clinical studies.

There are some limitations in this study. First, it is unclear whether an MEP recording time of 10 minutes is sufficient. When it was observed that the cranial environment did not change, and so as not to disturb the progress of the surgery, 10 minutes was the limit. In all three cases, MEP amplitudes reached a plateau a few minutes after the start of sevoflurane inhalation, but the amplitudes could be depressed further after 10 minutes. Second, because we recorded TCeMEP by suprathreshold stimulation, a different result could be provided if a stimulation condition was changed to supramaximal stimulation. Therefore, the monitoring of TCeMEP could be possible by changing the stimulating condition under a mixture of propofol and 0.5 MAC sevoflurane.

## Conclusions

We reported and estimated three cases in which changes over time of TCeMEP could be observed under anesthetic management using propofol and a low concentration (0.2 MAC and 0.5 MAC) of sevoflurane in neurosurgery. TCeMEP as an evaluation of motor function could be recorded under the condition of a combination of 0.2 MAC sevoflurane with propofol, but recording could be difficult with 0.5 MAC sevoflurane in propofol anesthesia. Further clinical studies are needed to elucidate the efficacy and safety of low-concentration sevoflurane inhalation in TIVA.
